# On Metastases in Malignant Disease

**Published:** 1907-10-26

**Authors:** 


### The Hospital

A Journal of

The Medical Sciences and Hospital Administration.

New Series. No. 31 Vol. II. [No. 1103, Vol. XLIII.] SATURDAY, OCTOBER 26, 1907.

### On Metastases in Malignant Disease.

There are many obscure and complex problems associated with the natural history of malignant growths, and, as is well known, these are at the present day being vigorously and systematically attacked by an energetic army of scientific workers. The investigations thus conducted appear in many instances to be far removed from the immediate claims of clinical work, and out of them has grown a language, or at least a terminology, that is almost a closed book to those unfamiliar with the technicalities of the laboratory. Yet no one acquainted with the history of scientific advance would stay the progress of work on the ground that it cannot show at once a utilitarian justification and defence. It is not thus that the greatest triumphs have been won. And now, as in former times, we must be content to let knowledge grow in whatever direction is possible, confident that sooner or later its value and application to affairs will be made manifest. The study of the so-called metastases or secondary developments from cancerous tumours may be placed among the subjects which thus appeal to the student of morbid structure and function. The circumstances which govern these developments, the channel or route of extension, and the relation of the metastatic distribution to the situation of the primary growth, are all questions making high appeal to the scientific pathologist. To the clinician such questions must have a much less attractive quality. His therapeutic powers, whether medical or surgical, are for the most part stayed when malignant metastases have once occurred. Some relief of suffering he may accomplish, but further than this his ambition will hardly soar.

Yet there are several circumstances in connection with metastases secondary to malignant tumours which present a high degree of clinical interest and importance. These are found more particularly in cases where the primary growth is either not within reach of observation, or, though observed, is misinterpreted. In such circumstances the secondary developments may be the first clinical events to attract attention, or at least to create anxiety and alarm. At first sight this would appear to be a position not likely to be frequently presented. Yet in one direction it is a comparatively common occurrence, and is familiar to every practitioner. Thus malignant disease of the liver is by no means one of the rarities of practice, and in the vast majority of cases it is a process secondary to a primary growth in some such situation as the stomach or rectum. Yet the clinical history is very often devoid of any hint of the existence of such primary growth, and in its practical aspect the case is merely one of hepatic disease. A less striking but somewhat similar example may be seen in cases where supra-clavicular glandular enlargement is an early expression of malignant disease of the œsophagus or other part of the upper portion of the alimentary canal. In both of these instances, but particularly in the former, it is the secondary or metastatic event which appears on the surface to be of chief clinical importance.

Some little time ago it was shown by Professor Osler that the order of events above described may be exactly repeated in cases of carcinoma of the breast. The original tumour may be so small as to escape detection, or, though recognised as a fact, its nature may be misunderstood. And this state of matters may exist until secondary extension becomes an urgent clinical fact. But there are other influences which tend to promote the misinterpretation of secondary malignant growths. One of these is the length of time that may elapse between the removal of the primary growth and the occurrence of metastases. This may be a matter of several years, and thus the true nature of the later developments may for a time be misunderstood. Still more important possibly is the fact that when such metastases occur in the central nervous system objective evidences of their presence may for a long time be altogether absent. Particularly is this true when the secondary tumours develop in the spinal canal. The evidence is clear that such tumours may for long periods of time cause the most extreme and severe pain without any disturbance of motion or sensation, without interference with the functions of the bladder or rectum, and without any alteration of the normal tendon phenomena. It is not surprising that in these circumstances the pains may be regarded as neuralgic or hysterical in nature, and may thus be dismissed in a more or less airy fashion. The event sooner or later passes a painful judgment on such a conclusion, but later wisdom is only a partial compensation for present blunders.

The moral is that with a history of a growth of possibly malignant origin the development of pains or other forms of discomfort, even in the absence of objective facts, must be regarded as highly suggestive of secondary developments in the central nervous system, and must demand a prognosis shadowed by suspicion. Further, what is true of an external tumour and its secondary consequences is equally true of malignant disease of the viscera and the metastases which may follow this. Carcinoma of the lung or bladder, for example, may be, like carcinoma of the breast, a comparatively obscure process until mischief follows in some remote part of the body. It is only if this truth is remembered that such secondary developments when they occur will receive a due measure of clinical justice.

